# RETRACTION: The Increased PTK7 Expression Is a Malignant Factor in Cervical Cancer

**DOI:** 10.1155/dim/9817180

**Published:** 2025-05-07

**Authors:** Disease Markers

RETRACTION: J.-J. Sun, H.-L. Li, S.-J. Guo, H. Ma, S.-J. Liu, D. Liu, and F.-X. Xue. “The Increased PTK7 Expression Is a Malignant Factor in Cervical Cancer,” *Disease Markers*, no. 2019 (2019): 1–10. https://doi.org/10.1155/2019/5380197.

The above article, published online on 3 March 2019 in Wiley Online Library (wileyonlinelibrary.com), has been retracted by John Wiley & Sons Ltd.

The retraction has been agreed following an investigation of the concerns raised by *Hoya camphorifolia* on PubPeer [1], which identified multiple instances of inappropriately overlapping figures.

Specifically:

  -Figure 2b: The Western Blot bands corresponding to PTK7 and *β*-actin expression in the MG-63 cells have been duplicated in 2 other papers with some rearrangements (Figure 2b in [2] and Figure 2b in [3]) and labelled as other proteins and cell types.
  -Figure 3a: The image of the cell cultures is identical to the 5637 cells shown in Figure 3a of [4], where it was labelled as a different cell type expressing a different target protein.
  -Figure 3d: The Western blot bands for PCNA and *β*-actin on the right side of the figure are identical to the bands corresponding to Ki67 and *β*-actin in the right panel of [5].
  -Figure 4d (left) /5d: The Western Blot images appear to be duplicated, while denoting different proteins.
  -Figure 5a: The second and third tumors in the top row as well as the first, second, and fourth tumors in the bottom row appear identical to the second and third tumors in the top row and the first 3 tumors in the bottom row in Figure 4a of [6]. The tumors also appear in Figure 5a of [7].
  -Figure 5b: The images of the two tumors above the graph have been duplicated in Figure 5b of [7] and Figure 4b of [8], while denoting different tissues.
  -Figure 5c: The immunohistochemistry panel in the top right of the figure has been duplicated in Figure 4c (right) of [8], and Figure 6d (right) of [9]. The image is labelled as a different tissue type in each manuscript.

As a result of the investigation, the data and conclusions of this article are considered unreliable.

Hong-Lin Li disagrees with this retraction. The other authors were informed of this decision but did not provide a response.
